# Pregabalin–Tolperisone Combination to Treat Neuropathic Pain: Improved Analgesia and Reduced Side Effects in Rats

**DOI:** 10.3390/ph16081115

**Published:** 2023-08-07

**Authors:** Nariman Essmat, Anna Rita Galambos, Péter P. Lakatos, Dávid Árpád Karádi, Amir Mohammadzadeh, Sarah Kadhim Abbood, Orsolya Geda, Rudolf Laufer, Kornél Király, Pál Riba, Zoltán S. Zádori, Éva Szökő, Tamás Tábi, Mahmoud Al-Khrasani

**Affiliations:** 1 Department of Pharmacology and Pharmacotherapy, Semmelweis University, 4 Nagyvárad tér, H-1089 Budapest, Hungary; nariman.gomaa@phd.semmelweis.hu (N.E.); galambos.anna@phd.semmelweis.hu (A.R.G.); karadi.david_arpad@med.semmelweis-univ.hu (D.Á.K.); mohammadzadeh.amir@med.semmelweis-univ.hu (A.M.); abbood.sarah@phd.semmelweis.hu (S.K.A.); kiraly.kornel@med.semmelweis-univ.hu (K.K.); riba.pal@med.semmelweis-univ.hu (P.R.); zadori.zoltan@med.semmelweis-univ.hu (Z.S.Z.); 2 Department of Pharmacology and Toxicology, Faculty of Pharmacy, Zagazig University, Zagazig 44519, Egypt; 3 Department of Pharmacodynamics, Semmelweis University, 4 Nagyvárad tér, H-1089 Budapest, Hungary; lakatos.peter.pal@semmelweis.hu (P.P.L.); geda.orsolya@semmelweis.hu (O.G.); laufer.rudolf@semmelweis.hu (R.L.); szoko.eva@semmelweis.hu (É.S.)

**Keywords:** neuropathic pain, allodynia, tolperisone, pregabalin, duloxetine, CSF glutamate content, synaptosome, neuronal glutamate release

## Abstract

The current treatment of neuropathic pain (NP) is unsatisfactory; therefore, effective novel agents or combination-based analgesic therapies are needed. Herein, oral tolperisone, pregabalin, and duloxetine were tested for their antinociceptive effect against rat partial sciatic nerve ligation (pSNL)-induced tactile allodynia described by a decrease in the paw withdrawal threshold (PWT) measured by a dynamic plantar aesthesiometer. On day 7 after the operation, PWTs were assessed at 60, 120, and 180 min post-treatment. Chronic treatment was continued for 2 weeks, and again, PWTs were measured on day 14 and 21. None of the test compounds produced an acute antiallodynic effect. In contrast, after chronic treatment, tolperisone and pregabalin alleviated allodynia. In other experiments, on day 14, the acute antiallodynic effect of the tolperisone/pregabalin or duloxetine combination was measured. As a novel finding, a single dose of the tolperisone/pregabalin combination could remarkably alleviate allodynia acutely. It also restored the neuropathy-induced elevated CSF glutamate content. Furthermore, the combination is devoid of adverse effects related to motor and gastrointestinal transit functions. Tolperisone and pregabalin target voltage-gated sodium and calcium channels, respectively. The dual blockade effect of the combination might explain its advantageous acute analgesic effect in the present work.

## 1. Introduction

Neuropathic pain (NP) is a debilitating chronic condition that results from disease, trauma, or dysfunction affecting the somatosensory neurons. Several mechanisms are involved in the development of NP; mechanisms that alter the balance between operating excitatory and inhibitory neurotransmitters at the spinal cord level are of importance [1]. The current pharmacological lines of therapy for NP encompass different drugs. First-line treatments include gabapentinoids affecting the high voltage-activated calcium channels hosting α2δ-1 subunits that are localized on the excitatory neurons on the dorsal horn of the spinal cord, and antidepressants that are non-selective or selective inhibitors of serotonin and noradrenaline reuptake, such as amitriptyline or duloxetine, respectively. The second-line treatments include the use of voltage-gated sodium channel inhibitors, such as topical lidocaine or drugs acting on other receptors, including tramadol, which simultaneously target both the noradrenergic and serotoninergic systems, yet activate opioid receptors [2,3]. The third-line treatments are strong opioids and subcutaneous botulinum toxin A. Furthermore, treatments include combination therapy, antiepileptic agents, selective serotonin reuptake inhibitors, NMDA receptor antagonists, and local capsaicin [3,4,5,6,7]. All the above-mentioned treatment approaches consist of different drugs of various pharmacodynamic targets, reflecting the complex nature of the pathophysiology of neuropathic pain [8,9,10,11,12]. Despite these treatment options, the current drugs used to treat NP cause side effects that result in dose escalation being practically impossible. To solve this problem, multimodal analgesia containing two or more analgesics at lower doses may provide additive or synergistic effects of increased efficacy, and decreased side effects compared to the single therapy. Several human studies have followed this strategy; among them is the combination of gabapentinoids with tricyclic antidepressants, such as nortriptyline or opioid analgesics, to manage post-herpetic neuralgia or painful diabetic polyneuropathy [13,14,15]. In these studies, the combination offers better analgesia as compared to the use of single-drug therapy. However, the adverse effects, such as dry mouth and constipation, among others, caused by the anticholinergic and opioid constituents of the combination were higher compared to gabapentinoids medication alone [16]. Another study showed that treating NP of diabetic patients with a combination composed of oxycodone and gabapentin did not worsen the commonly observed opioid-induced side effects, which supports the use of this combination for neuropathic pain control [17]. A discrepancy between studies has been reported regarding the analgesic effect of the combination of oxycodone and gabapentinoids, such as pregabalin, where a small dose of oxycodone has failed to enhance pregabalin’s ability to relieve pain in patients with either painful diabetic neuropathy or post-herpetic neuralgia [18]. Numerous preclinical studies have also been carried out to evaluate the combination of different drugs that were used to manage human neuropathic pain. In neuropathic rats that underwent spinal nerve ligation, Matthews and Dickenson (2002) showed that, in contrast to morphine, gabapentin’s inhibitory effect is increased in the dorsal horn neuronal response following its systemic administration [19], yet the response to morphine was diminished. The pregabalin–carbamazepine (sodium channel blocker) combination was found to produce a synergistic antiallodynic effect in the spinal nerve ligation model in rats. In this study, the antiallodynic effect was only seen when the drugs were combined in doses that exceeded ED75 values, although the side effects were not studied [20]. For more details on the analgesic and side effects of drug combinations being used to treat NP, see [21].

Tolperisone is a centrally acting muscle relaxant used clinically for various conditions, such as painful reflex muscle spasms and post-stroke spasticity [22,23]. Its mechanism of action has been suggested to include the blocking of sodium and calcium channels [24,25,26]. Recent research carried out by our group reported that acute oral tolperisone administration can induce a measurable acute antinociceptive effect against mechanical allodynia in neuropathic rats [27].

The present preclinical work includes a comparative study of drugs used to ameliorate human neuropathic pain by different mechanisms of action, such as pregabalin, duloxetine, and carbamazepine. A multimodal approach to manage NP has long been appreciated; thus, the present study also intended to explore the potential relevance of tolperisone in tactile allodynia treatment, particularly when combined with pregabalin, a combination that has not been tested before. In this regard, we hypothesized that the use of two mechanistically different analgesics, tolperisone (sodium channel blocker) with pregabalin (calcium channel blocker), at sub-analgesic doses may provide superior analgesia with a better adverse effect profile in tactile allodynia induced by partial sciatic nerve ligation or streptozotocin (STZ)-induced peripheral neuropathic pain in rats compared to each single drug to identify new combinational therapies for neuropathic pain treatment in future clinical trials. The study also includes experiments on the impact of the co-administration of tolperisone and duloxetine on pSNL-induced NP. Finally, we assessed the motor and gastrointestinal side effects of the combination-based promising analgesic approach to treat NP.

## 2. Results

### 2.1. Chronic Treatment Is Essential for Both Tolperisone and Pregabalin to Alleviate Tactile Allodynia Evoked by Partial Sciatic Nerve Ligation (pSNL)

**Figure 1:**
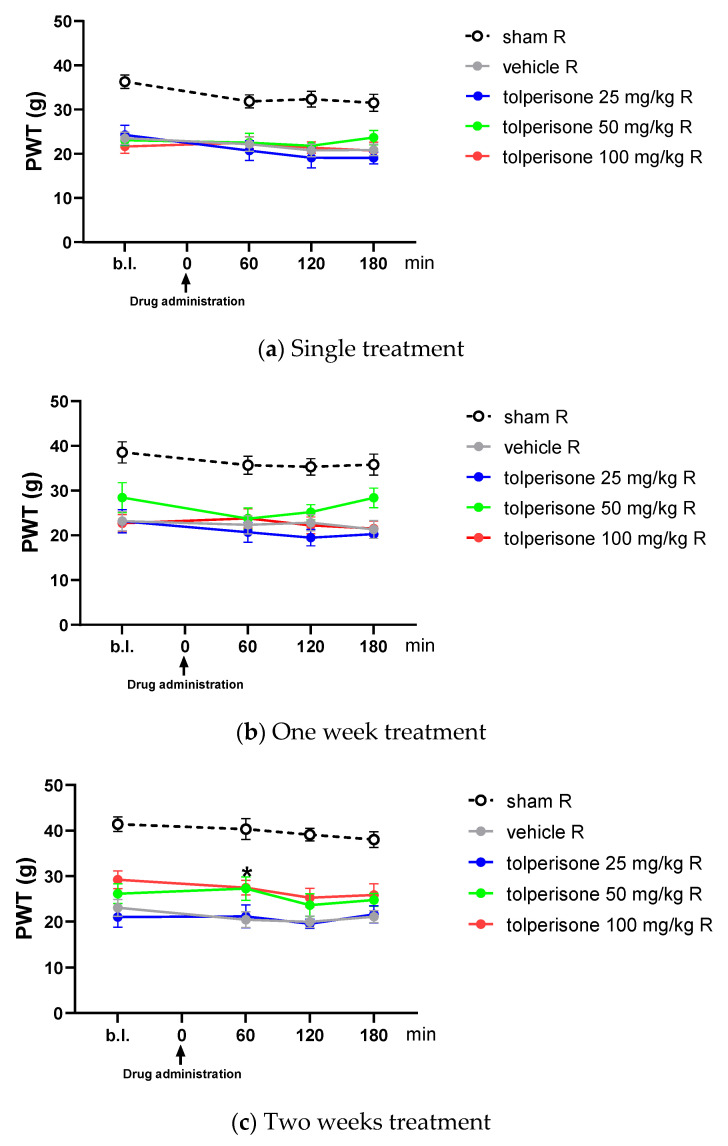
The antiallodynic effect of tolperisone on pSNL evoked allodynia. The PWTs were measured postoperatively on day 7 prior to and after a single treatment (panel (**a**)), on day 14 after 1 week of chronic treatment (panel (**b**)), and on day 21 after 2 weeks of chronic treatment (panel (**c**)). Tactile allodynia was measured by DPA at 60, 120, and 180 min after oral treatment. Data are shown as the mean ± SEM of 8–13 animals per group. * *p* < 0.05 statistically significant compared to the vehicle-treated group at the indicated time points after treatment (two-way ANOVA followed by Dunnett’s post-hoc test). Baseline (b.l.): was measured before the first treatment. Single treatment: was measured after acute administration on day 7 after the operation.

**Figure 2:**
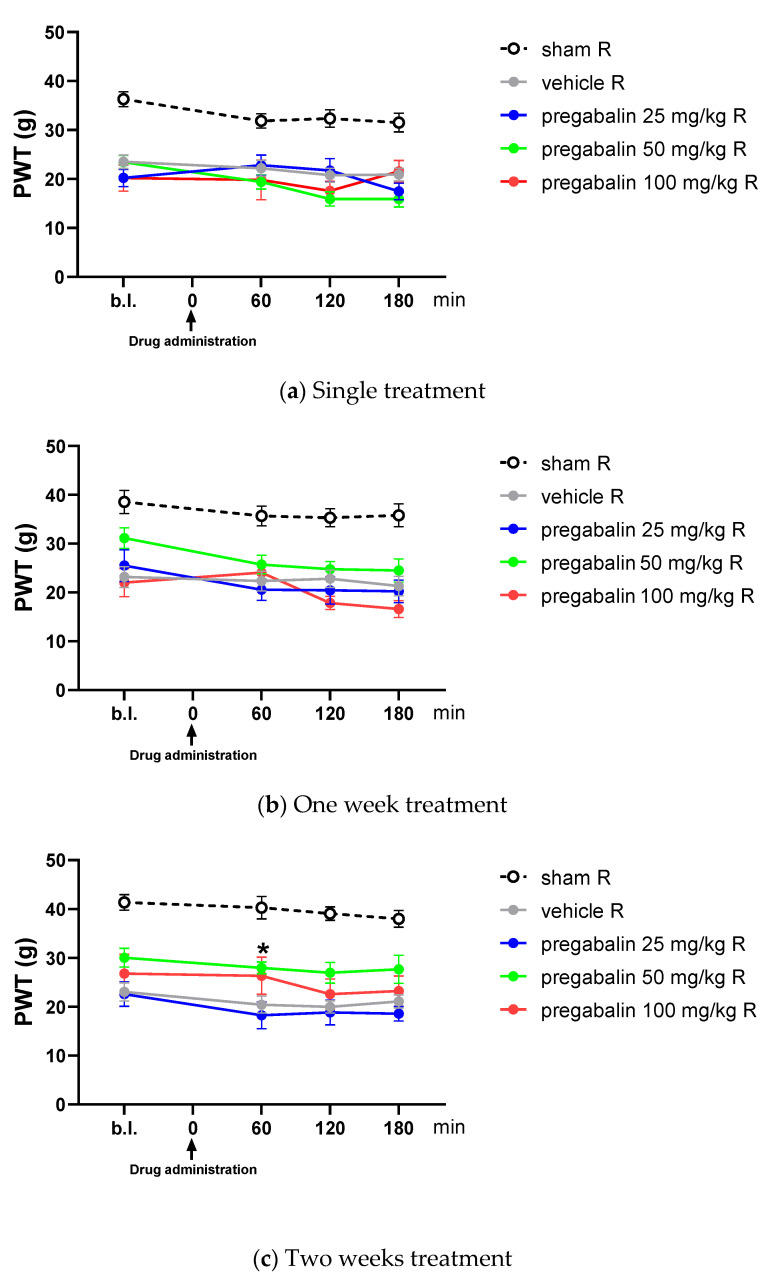
The antiallodynic effect of pregabalin on pSNL evoked allodynia. The PWTs were measured on day 7 prior to and after a single treatment (panel (**a**)), on day 14 (1 week treatment) (panel (**b**)), and on day 21 (2 weeks treatment) (panel (**c**)) after the operation. Tactile allodynia was measured by DPA at 60, 120, and 180 min after oral treatment. Data are shown as the mean ± SEM of 6–13 animals per group. * *p* < 0.05, statistically significant compared to the vehicle right (operated) paw at the indicated time points after treatment (two-way ANOVA followed by Dunnett’s post-hoc test). Baseline (b.l.): was measured before the first treatment. Single treatment: was measured after acute administration on day 7 after the operation.

Figure 1 and Figure 2 depict the effect of orally administered tolperisone or pregabalin (25, 50, and 100 mg/kg) in pSNL-induced tactile allodynia in rats on day 7 (single treatment), 14 (1 week treatment), and 21 (2 weeks treatment) after the operation at 60, 120, and 180 min. Tactile allodynia was indicated by a decrease in the rat paw withdrawal threshold (PWT) measured by a dynamic plantar aesthesiometer (DPA).

In this series of experiments, we intended to measure the acute effect of oral (25, 50, and 100 mg/kg) tolperisone or pregabalin on the developed tactile allodynia of rats with pSNL on day 7, as well as of rats that were treated for 2 consecutive weeks (Figure 1 and Figure 2). As shown in Figure 1a,b, oral test doses of tolperisone failed to produce a significant antiallodynic effect either after acute treatment or 1 week of chronic treatment, respectively. After 2 weeks of treatment, following oral administration, 100 mg/kg of tolperisone showed a significant effect against the developed tactile allodynia, 60 min after oral administration (Figure 1c) compared to the vehicle-treated group (two-way ANOVA: F (treatment group; 4, 45) = 25.41, *p* < 0.0001, Dunnett’s post-hoc test: *p* = 0.0260).

Similar to tolperisone, pregabalin in test doses was ineffective in alleviating rat tactile allodynia following acute treatment or 1 week of chronic treatment (Figure 2a,b). However, 2 weeks of consecutive treatment with 50 mg/kg pregabalin significantly alleviated tactile allodynia 60 min after oral administration (two-way ANOVA: F (treatment group; 4, 39) = 23.91, *p* < 0.0001, Dunnett’s post-hoc test: *p* = 0.0080) when compared to the vehicle-treated group (Figure 2c).

**Figure 3:**
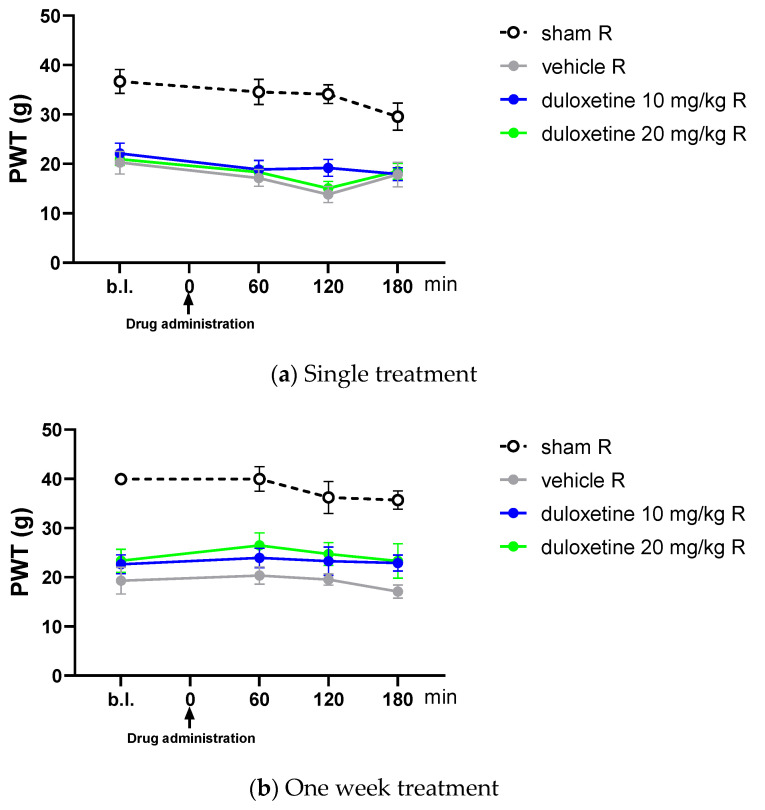

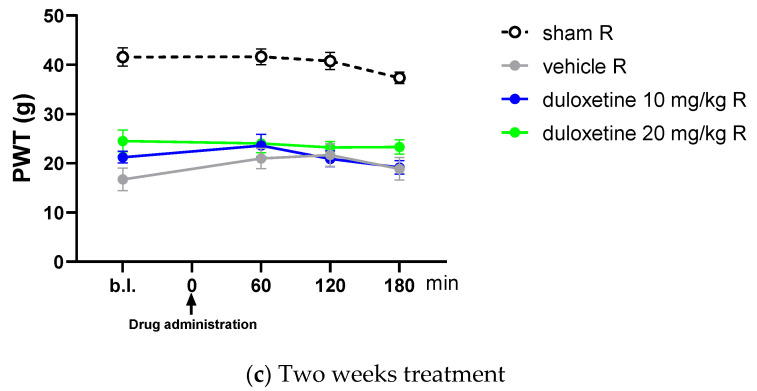
The antiallodynic effect of duloxetine on pSNL evoked allodynia. The PWTs were measured on day 7 prior to and after a single treatment (panel (**a**)), on day 14 (1 week treatment) (panel (**b**)), and on day 21 (2 weeks treatment) (panel (**c**)) after the operation. Tactile allodynia was measured by DPA at 60, 120, and 180 min after oral treatment. Data are shown as the mean ± SEM of 5–7 animals per group. *p* < 0.05, statistically significant compared to the vehicle right (operated) paw at the indicated time points after treatment (two-way ANOVA followed by Dunnett’s post-hoc test). Baseline (b.l.): was measured before the first treatment. Single treatment: was measured after acute administration on day 7 after the operation.

On the other hand, duloxetine in test doses was ineffective in alleviating rat tactile allodynia following acute or chronic treatment compared to vehicle-treated rats (Figure 3a–c).

### 2.2. Acute Oral Co-Administration of Tolperisone with Pregabalin but Not Duloxetine Alleviates Tactile Allodynia of Rats with Neuropathic Pain Evoked by pSNL

**Figure 4:**
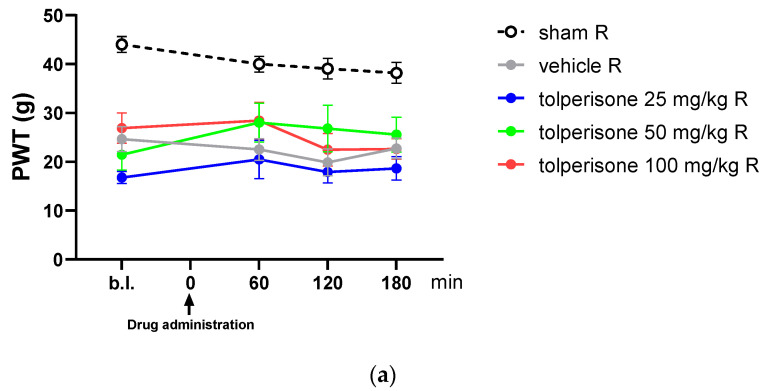

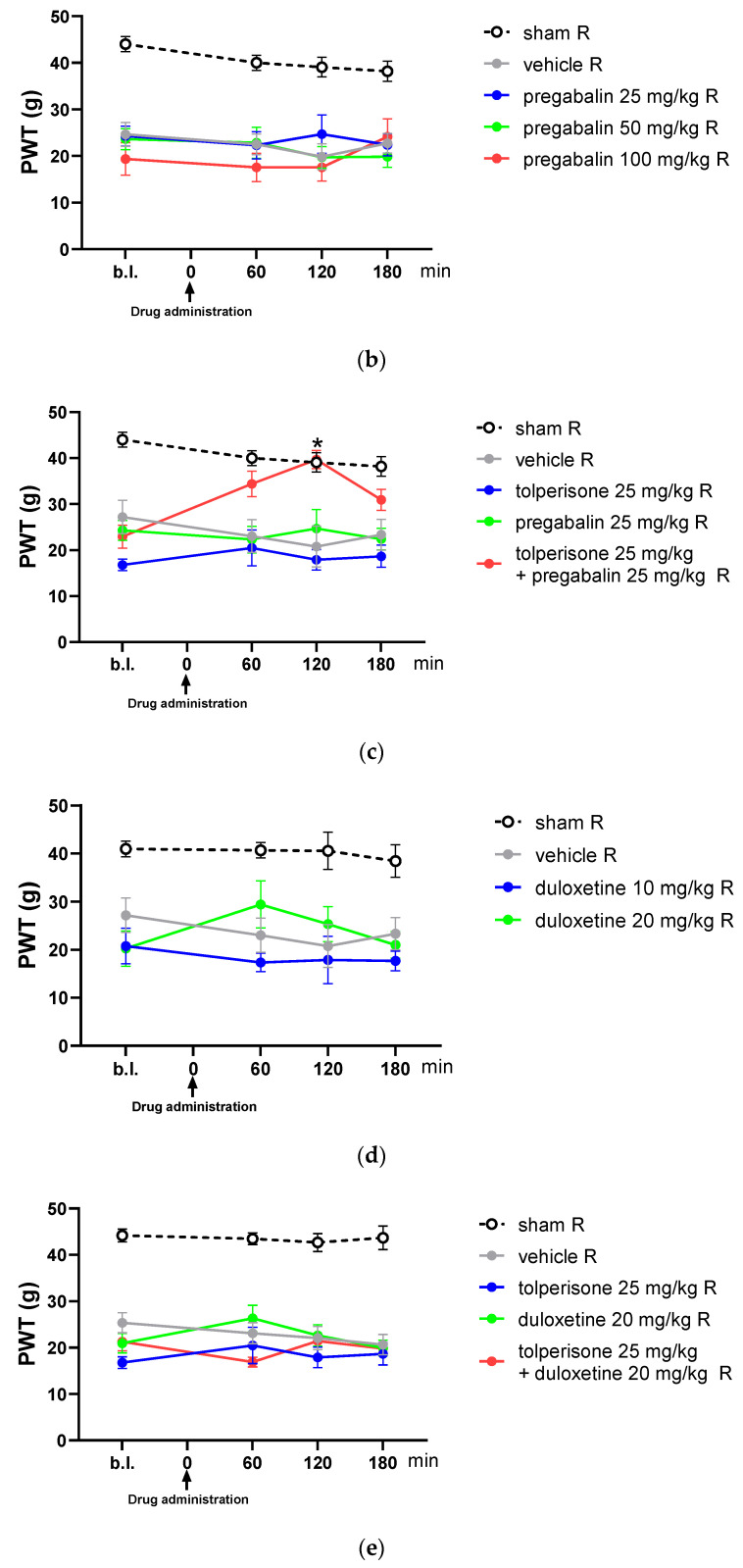
The acute antiallodynic effect of tolperisone (panel (**a**)), pregabalin (panel (**b**)), a combination of tolperisone and pregabalin (panel (**c**)), duloxetine (panel (**d**)), and a combination of tolperisone and duloxetine (**e**) on the pSNL evoked allodynia. The PWT was measured on day 14 after the operation. Tactile allodynia was measured by DPA at 60, 120, and 180 min after acute oral treatment. Data are shown as the mean ± SEM of 6–8 animals (panel (**a**)), 8 animals (panel (**b**)), 5–8 animals (panel (**c**)), 4–5 animals (panel (**d**)), and 5–8 animals (panel (**e**)) per group. * *p* < 0.05, statistically significant compared to the vehicle right (operated) paw at the indicated time points after treatment (two-way ANOVA followed by Dunnett’s post-hoc test). Baseline (b.l.): was measured before the treatment.

In this phase of the study, we followed a strategy of multimodal analgesia namely combining pregabalin and tolperisone. Thus, the pregabalin and tolperisone combination was investigated in animals showing allodynia two weeks after pSNL (Figure 4). As stated above, treatment with tolperisone or pregabalin at a dose of 25 mg/kg did not cause significant analgesic effects after either acute or chronic oral administration (Figure 1 and Figure 2). Furthermore, Figure 4 showed that a single treatment with tolperisone (Figure 4a), pregabalin (Figure 4b), or duloxetine (Figure 4d) failed to induce significant effects in the PWTs of all treatment groups at 60, 120, and 180 min. Interestingly, pregabalin and tolperisone (25 mg + 25 mg) significantly alleviated the tactile allodynia of rats with NP at 120 min after acute oral administration compared to the vehicle (two-way ANOVA: F (treatment group; 4, 28) = 17.81, *p* < 0.0001, Dunnett’s post-hoc test: *p* = 0.0266), tolperisone, or pregabalin-treated groups (Figure 4c). In contrast, the combination of tolperisone and duloxetine failed to attenuate the rat tactile allodynia following acute oral administration at the doses and time points indicated in Figure 4e.

### 2.3. The Impact of Pregabalin and Tolperisone on Peripheral Neuropathic Pain of Diabetic Rats after Acute Administration

**Figure 5:**
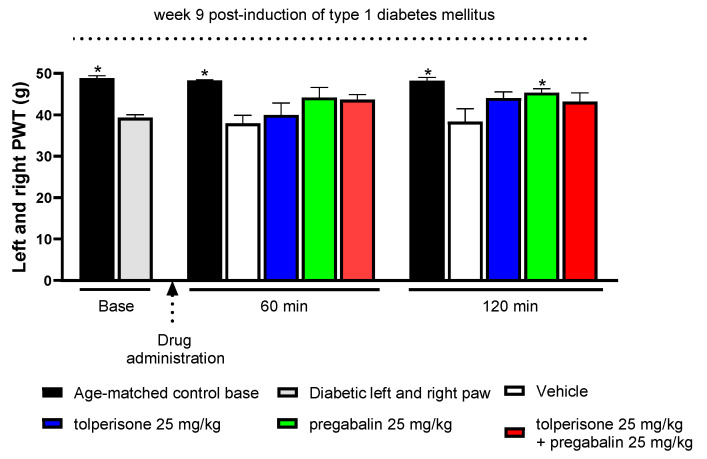
The acute antiallodynic effect of tolperisone and pregabalin on type 1 diabetes evoked tactile allodynia. The left and right PWT was measured in week 9 after diabetes induction. Tactile allodynia was measured by DPA at 60 and 120 min after acute oral treatment. Data are shown as the mean ± SEM of 3–5 animals per group. * *p* < 0.05, statistically significant compared to the vehicle-treated groups (one-way ANOVA followed by Dunnett’s post-hoc test). The baseline was measured before treatment.

**Figure A1:**
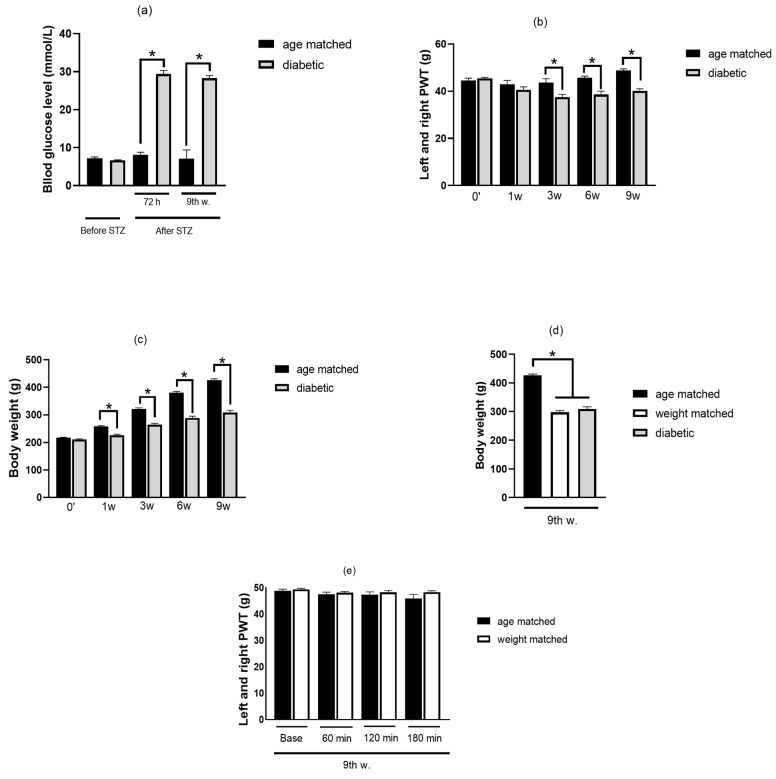
Blood glucose level (panel (**a**)), left and right PWT of age-matched and diabetic animals (panel (**b**)), the body weight of age-matched and diabetic animals (panel (**c**)), the body weight of age and weight-matched, and diabetic animals at 9 weeks (panel (**d**)), left and right PWT of age and weight-matched animals at 9 weeks (panel (**e**)). Data are shown as mean ± S.E.M. of 6 age-matched, 6 weight-matched, and 20 diabetic animals. * *p* < 0.05 statistically significant compared to the age-matched animals. Data were analyzed by unpaired *t*-test for panels (**a**–**c**) and (**e**) and one-way ANOVA followed by Dunnett’s post-hoc test, panel (**d**).

Based on the promising effect obtained in the mononeuropathic pain model, we extended our investigations to assess the antiallodynic effect of the tolperisone–pregabalin combination (25 and 25 mg/kg) in streptozotocin (STZ)-induced diabetic polyneuropathy. In accordance with our previous study [28], STZ treatment evoked a significant increase in the blood glucose level observed 72 h after 60 mg/kg STZ intraperitoneal injection compared to age-matched control animals that were maintained over the entire experiment (9 weeks), (see Figure A1a). Additionally, 3 weeks after STZ injection, the onset of tactile allodynia was indicated by a significant decrease in left and right PWTs that was maintained over the whole period (see Figure A1b). We determined the effect of the individual components of the combination (pregabalin and tolperisone both at 25 mg/kg), as well as the combination itself, 9 weeks following STZ treatment at 60 and 120 min after oral administration. Acute treatment with 25 mg/kg pregabalin produced a significant antiallodynic effect after 120 min (one-way ANOVA: F (11, 31) = 7.167, *p* < 0.0001, Dunnett’s post-hoc test: *p* = 0.0139); however, tolperisone or the combination could induce only a tendentious effect (Figure 5). Significant changes between the body weight of diabetic rats and the nondiabetic age-matched animals were observed after 1 week and thereafter (see Figure A1c). In order to justify our results, weight-matched animals were also used in order to assess the impact of body weight on the PWT (see Figure A1d). Since the weight-matched animals showed a PWT similar to that of the age-matched animals at 9 weeks at all tested time points (see Figure A1e), the age-matched animals were used for comparison in this experiment.

### 2.4. Effects of Acute Treatment with Tolperisone, Pregabalin, or their Combination on CSF Glutamate Content in Rats with pSNL-Induced Neuropathic Pain

**Figure 6:**
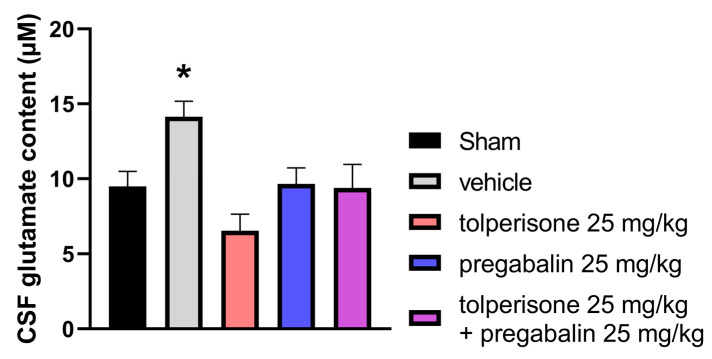
CSF glutamate content of mono-neuropathic (pSNL) and sham operated rats after acute treatment with orally administered tolperisone (25 mg/kg), pregabalin (25 mg/kg), or their combination (both at 25 mg/kg) on day 14 after pSNL operation. Data are shown as mean ± SEM of n = 4–21 animals per group. * *p* < 0.05 vs. other groups (one-way ANOVA followed by Dunnett’s post-hoc test).

Samples of cerebrospinal fluid (CSF) were taken from mono-neuropathic animals 14 days after pSNL operation, and their glutamate content was assessed by capillary electrophoresis. The vehicle treated mono-neuropathic animals showed a significant increase in the CSF glutamate concentration compared to the sham operated group (one-way ANOVA: F (4, 64) = 6.435, *p* = 0.0002, Dunnett’s post-hoc test: *p* = 0.0032). Tolperisone, pregabalin, or their combination significantly inhibited the nerve injury induced elevation of the CSF glutamate content and normalized it to the level of the sham operated group (Figure 6).

### 2.5. Effects of Treatment with Tolperisone, Pregabalin, or Their Combination on 4-Aminopyridine-Induced Glutamate Release from Rat Synaptosomes

**Figure 7:**
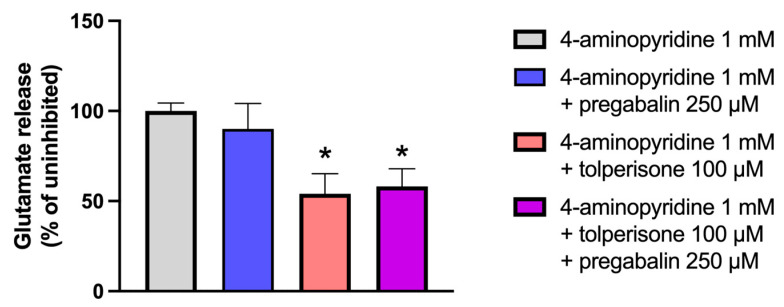
Effect of tolperisone (100 μM), pregabalin (250 μM), or their combination on glutamate release from rat brain synaptosomes induced by 1 mM 4-aminopyridine. Drugs were administered as a pretreatment 20 min prior to stimulation. The concentration of released glutamate was measured 6 min after stimulation. All data points were normalized using the unstimulated, baseline release and presented as % of the stimulated glutamate release in the absence of test compounds (gray bar). All columns show the mean of glutamate release ± SEM in % in the indicated groups. * *p* < 0.05 vs. stimulated glutamate release by 1 mM 4-aminopyridine alone and treatment groups (one-way ANOVA followed by Dunnett’s post-hoc test). In each treatment group, 4–13 parallel experiments were used.

The effect of tolperisone (100 μM), pregabalin (250 μM), or their combination on depolarization-induced glutamate release from rat brain synaptosomes was measured to better understand their probable mode of action. 4-aminopyridine, a K^+^-channel inhibitor, was used to induce depolarization and subsequent neurotransmitter release [29]. 4-aminopyridine-induced transmitter release depends on the activation of sodium and calcium channels [30] and was blocked by tolperisone but not by pregabalin in accordance with our previous results [27]. Here, the combination was found to also significantly inhibit glutamate release induced by 4-aminopyridine (one-way ANOVA: F (3, 24) = 8.686, *p* = 0.0004, Dunnett’s post-hoc test: 100 μM tolperisone, *p* = 0.0012; 100 μM tolperisone and 250 μM pregabalin, *p* = 0.0016, Figure 7).

### 2.6. The Impact of Pregabalin, Tolperisone, and Pregabalin/Tolperisone Combination on Motor Dysfunction and Coordination Imbalance in Naïve Rats

**Figure 8:**
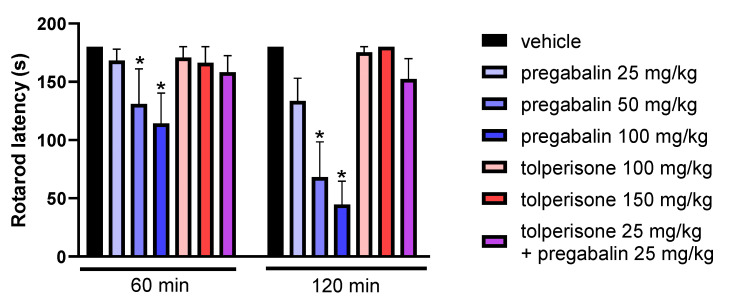
Effect of acute oral administration of tolperisone (100 and 150 mg/kg), pregabalin (25, 50, and 100 mg/kg), and the combination of tolperisone and pregabalin (both at 25 mg/kg) or vehicle at 60 and 120 min on motor coordination and balance of animals. Columns show the time latency in the rotarod assay. Data are shown as the mean ± SEM of 5–19 animals per group, measured at the peak effect of test compounds. * *p* < 0.05 statistically significant compared to the vehicle (one-way ANOVA followed by Dunnett’s post-hoc test).

Acute oral pregabalin (50 and 100 mg/kg) but not tolperisone (100 and 150 mg/kg) treatments negatively influenced rats’ motor coordination and balance, as indicated by a significant decrease in time to stay on a rotating rod (one-way ANOVA: F (13, 84) = 11.12, *p* < 0.0001, Dunnett’s post-hoc test: 50 mg/kg, 60 min, *p* = 0.0326; 100 mg/kg, 60 min, *p* = 0.0010, 50 mg/kg; 120 min, *p* < 0.0001, 100 mg/kg; 120 min, *p* < 0.0001, Figure 8). On the other hand, pregabalin, at a dose of 25 mg/kg, did not elicit a change in rats’ motor coordination and balance (Figure 8). The treatment with the combination of pregabalin and tolperisone (both at 25 mg/kg) failed to elicit alteration in rats’ motor coordination and balance compared to the vehicle-treated group either 60 or 120 min after oral administration (Figure 8).

### 2.7. The Impact of Pregabalin, Tolperisone, and Pregabalin/Tolperisone Combination on Gastrointestinal (GI) Transit in Naïve Rats

**Figure 9:**
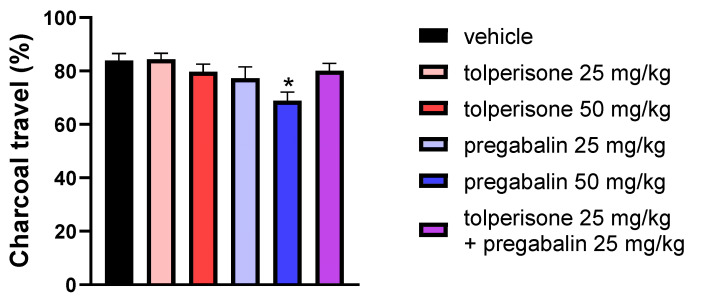
Effect of acute oral administration of tolperisone (25 and 50 mg/kg), pregabalin (25 and 50 mg/kg), and the tolperisone and pregabalin combination (both at 25 mg/kg) on the GI transit of naïve animals 30 min after a charcoal meal. Columns represent the charcoal travel (%) in the charcoal meal test. Data are shown as the mean ± SEM of 5–6 animals per group, measured at the peak effect of test compounds. * *p* < 0.05 statistically significant compared to the vehicle (one-way ANOVA followed by Dunnett’s post-hoc test).

Acute oral administration of tolperisone (25 and 50 mg/kg), pregabalin (25 mg/kg), and a combination of tolperisone and pregabalin (both at 25 mg/kg) failed to exhibit delays in the GI transit of a charcoal suspension in rats. However, acute pregabalin treatment with a dose of 50 mg/kg induced a moderate but significant delay in the GI transit of a charcoal suspension in rats compared to the vehicle (one-way ANOVA: F (5, 29) = 3.297, *p* = 0.0177, Dunnett’s post-hoc test: *p* = 0.0110, Figure 9).

## 3. Discussion

It is of great clinical significance to develop a new medication or a novel combination treatment approach to treat NP. The difficulty in treating NP stems from the diversity of etiologies (injuries, illnesses, drugs, etc.) and the intricacy of the underlying mechanisms. All these factors contribute to the poor effects of the current mono or combination therapies to effectively manage NP symptoms. In this regard, the current therapeutic strategies of NP continue to be unsatisfactory because they have a low efficacy of pain inhibition, delayed onset of action, and deleterious adverse effects. Thus, an effective mono or combination therapy with a significant analgesic effect, fast onset of action, and a good safety profile is desperately needed [31,32]. In this context, the present study principally intended to investigate the antiallodynic effect of tolperisone compared to pregabalin or duloxetine as monotherapy. Of importance, the efficacy of combinations composed of either tolperisone with pregabalin or duloxetine was also studied in the same NP model. In our previous work, we have only shown that tolperisone or pregabalin alone acutely inhibit the developed mechanical allodynia. However, herein, we applied another measurement approach, namely DPA, which is designed to determine more localized tactile allodynia in a small dynamic range. Furthermore, the effect of long-term treatment was also assessed. Similar to our previous work, the possible mechanism for inhibiting tactile allodynia was also investigated. We have also extended our investigations to assess the impact of the tolperisone–pregabalin combination on another neuropathic pain type, polyneuropathic pain evoked by type 1 diabetes. Finally, the possible effect of the promising combination on motor coordination and balance as well as gastrointestinal transit was assessed.

Mono-neuropathic pain was induced by pSNL in rats based on the Seltzer method. In comparison studies, the evolution of the antiallodynic effect of test compounds was carried out acutely and on day 7 or 14 post-operation. In experiments intended to evaluate the effect of chronic treatments, the treatment was initiated on day 7 after the operation and was continued for 14 days, and the antiallodynic impact was assessed on the 14th and 21st days after the operation. The suitability of the time periods for undertaking the treatment and pain assessment based on the present and previous studies has established that pSNL evokes stable tactile allodynia within 1 week following nerve ligation and lasts for at least 4 weeks [33,34]. Herein, we used this rat model to show the effect of test compounds and combinations against developed tactile allodynia within the described test period; the investigation was started on day 7 and ended before day 28, enabling us to avoid factors that may disturb the quality and productivity of the study. The results obtained from the rat mono-neuropathic pain model indicate that acute tolperisone treatment failed to alleviate tactile allodynia. This ineffectuality was also seen following acute treatment with pregabalin or duloxetine, two medications being used as first-line therapies for NP [35,36]. As already noted, the lack of an acute antiallodynic effect is in contrast with our previous results where mechanical allodynia was attenuated by acute tolperisone or pregabalin treatment, although the applied assay was different, as mentioned above [27]. To explain this, in our previous study, the antiallodynic effect of tolperisone or pregabalin was determined by the Randall–Selitto assay, which has been developed to measure the antinociceptive effect of test drugs on the pain thresholds evoked by mechanical pressure stimulation, although it can be considered a complement to cutaneous mechanical hyperalgesic assays [37]. On the other hand, we applied DPA, which is generally used to assess cutaneous mechanical hyperalgesia by applying filament stimuli to the plantar surfaces of the hind paws. It means that DPA likely detects tactile allodynia that needs long-term drug treatment to alleviate it. The difference between DPA and Randall–Selitto in terms of the dose of the drug administered was also observed previously by our group [38], namely, much higher analgesic doses are needed when DPA was used. Indeed, reports on the onset of the antiallodynic effect of pregabalin and duloxetine are contradictory [39,40,41,42]. For instance, applying a similar assay for at least 3 days was required for oral pregabalin to produce am antiallodynic effect in mice with NP evoked by cuffing the main branch of the sciatic nerve [43]. In addition, the analgesic assay, the route of administration, and particularly the type of NP are the main determinant factors in drawing a consensus about the analgesic effects of pregabalin and duloxetine, among others [15,44,45,46,47,48]. However, after chronic treatment, both tolperisone and pregabalin were able to elicit an antiallodynic effect. In fact, several preclinical and clinical studies have shown a similar lag time for pregabalin [49,50,51], but not for tolperisone. To the best of our knowledge, tolperisone has not been evaluated for potential analgesia in rats with preexisting tactile allodynia. This result raises a promising possibility for repurposing tolperisone for NP. With respect to duloxetine, despite the previous positive results, no significant antiallodynic effect could be measured either after acute or chronic oral treatment for 1 or 2 weeks [5]. Duloxetine is a serotonin and noradrenaline reuptake inhibitor; independent of its effect on depression, it alleviates allodynia in diabetic neuropathy. In this regard, spinal serotonin and noradrenaline play an important role in pain transmission. Pharmacological studies with serotonin and noradrenaline reuptake inhibitors have shown facilitatory or inhibitory effects on the descending serotonergic pathways that play a crucial role in neuropathic pain. This controversial or poor effect of serotonin has been attributed to the applied stimuli, the variable methods, and the timing of pain measurement [52,53].

**Figure A2:**
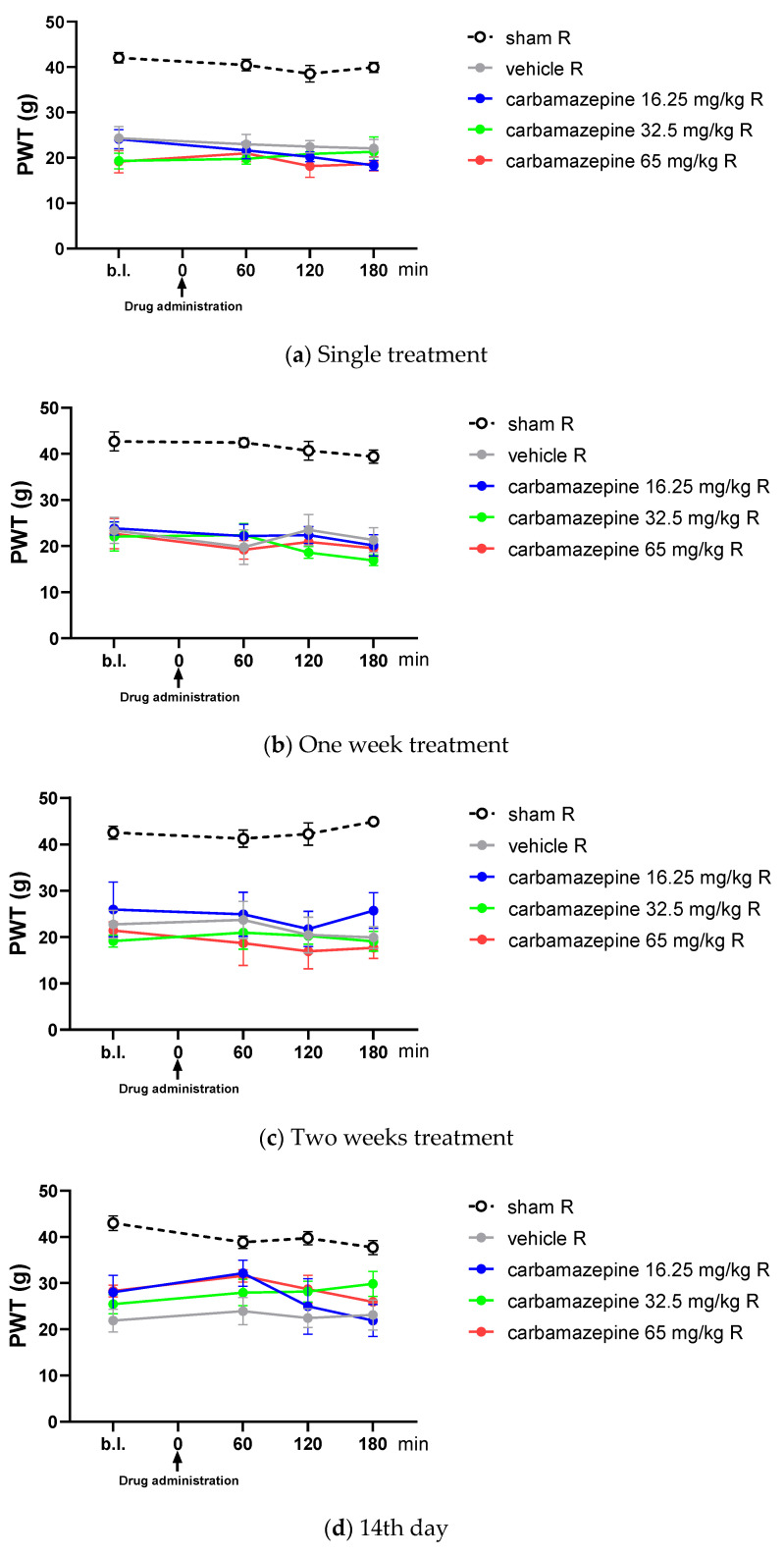
The antiallodynic effect of carbamazepine on pSNL evoked allodynia. The PWTs were measured on day 7 prior to and after a single treatment (panel (**a**)), on day 14 (1 week treatment) (panel (**b**)), on day 21 (2 weeks treatment) (panel (**c**)), and on day 14 after a single treatment (panel (**d**)) after the operation. Tactile allodynia was measured by DPA at 60, 120, and 180 min after oral treatment. Data are shown as the mean ± SEM of 6–7 animals (panels (**a**–**c**)) and 4–13 animals (panel (**d**)) in each group. *p* > 0.05, statistically not significant: treated groups versus the vehicle right (operated) paw at the indicated time points after treatment (two-way ANOVA followed by Dunnett’s post-hoc test). Baseline (b.l.): was measured before the first treatment. Single treatment: was measured after acute administration on day 7 after the operation.

Despite the discrepancy in the onset of action of the tested drugs, the essential finding of the present study is that the tolperisone/pregabalin, but not tolperisone/duloxetine, combination elicited a remarkable acute effect on the pSNL-induced allodynia at day 14 following operation. This result, to our best knowledge, is the first one to demonstrate the acute tactile antiallodynic efficacy of a low dose tolperisone–pregabalin combination in rat models of neuropathic pain. In addition, the effect of the combination was not associated with motor dysfunction or GI transit-related side effects. Indeed, the analgesic efficacy and safety of pregabalin alone or in combination with several drugs, but not with tolperisone, have been previously investigated in several clinical and preclinical studies for neuropathic pain treatment. Pregabalin and tolpersisone exert their analgesic effects against NP by inhibiting voltage-gated calcium channels hosting the α2-δ subunit and different voltage-dependent sodium channels, respectively [14,15,24,27]. Inhibition of these channels causes a reduction in calcium influx, neurotransmitter release, and, as a result, total neuronal excitability. It is worth noting that these channels are targets for first-line medications currently used to treat NP of different entities, including peripheral mono-neuropathic pain among others [15,54,55,56,57]. In addition, tolperisone has been reported to inhibit muscle spasms with an advantageous side-effect profile; it is devoid of the central side effects of the other centrally acting skeletal muscle relaxants. This property encouraged us to investigate its effect once combined with pregabalin. Voltage-gated sodium channel blockers, such as carbamazepine, are among the medications that are prescribed to treat NP [58,59]. The effect of carbamazepine either alone or combined with different drugs was studied in different neuropathic conditions. Fox et al. has reported that a single treatment with carbamazepine was ineffective against mechanical hyperalgesia or tactile allodynia in rats; however, it reversed mechanical hyperalgesia in guinea-pig NP evoked by pSNL [41,60]. This indicates that there is an overlap between the previously reported and present study regarding acute treatment with tolperisone or carbamazepine (see Figure A2a–d). With respect to the combination, it was shown that a gabapentin and carbamazepine combination induced synergistic analgesia compared to single drug administration, as indicated by high latency in the hot plate test in diabetic neuropathic rats [61]. Further, the pregabalin/carbamazepine combination was found to produce a synergistic antiallodynic effect in the rat spinal nerve ligation model. In fact, the antiallodynic effect was only seen when the drugs were combined in doses that exceeded the ED75 values, although, the side effects were not studied [20]. In fact, in our present study, the examined doses of tolperisone, pregabalin, or duloxetine that were shown to produce analgesia after chronic treatment are higher than the applied doses in the combination. It is worth noting that several preclinical and clinical studies have focused on the efficacy of carbamazepine, gabapentin, and pregabalin in managing trigeminal neuralgia, which is not the object of the present work [62,63,64,65,66]. Fortunately, the present study proceeded to identify combination therapy as having higher efficacy and fewer side effects. With respect to side effects, more than 50% of patients taking pregabalin experience considerable unwanted effects, most notably, excessive sedation [67]. Based on the above-mentioned literature of previous work and our present results, the tolperisone/pregabalin combination might be of clinical value, opening a possibility of repurposing.

It has been well established that rat NP evoked by peripheral nerve injury initiates both peripheral and central sensitization that concomitantly occurs with an imbalance between spinal excitatory and inhibitory systems [1]. An increase in spinal glutamate levels has been described as one of the major contributors to the mechanism responsible for the development of NP. Indeed, drugs that inhibit NMDA receptors are known to be among the effective management strategies for chronic pain [1,68]. Therefore, we also measured the CSF glutamate content, which was found to be increased in rats with NP. The inhibition of glutamatergic activity is one of the explanations proposed by researchers for the inhibition of mono and polyneuropathic pain [27,69,70,71,72,73]. However, the contribution of the glutamatergic system to the pathophysiology of NP is more complex than the simple thought of enhancing or decreasing the glutamatergic system.

We have also assessed the impact of the tolperisone–pregabalin combination on polyneuropathic pain evoked by type 1 diabetes. In this series of experiments, pregabalin has been shown to be a more efficacious analgesic than the combination that proved to be superior in mono-neuropathic pain, namely in the pSNL model. This effect of pregabalin was not surprising as several preclinical and clinical data support its effectiveness in diabetic neuropathy [74,75,76]. With respect to tolperisone, further future studies are needed to fully characterize its antiallodynic effect, but the present results suggest that it is most effective in nerve injury-induced NP, particularly when combined with pregabalin.

The major limitation of the present work was that in the diabetes-induced polyneuropathic pain model tolperisone, pregabalin, and their combination were only tested at a dose of 25 mg/kg, which was effective in nerve injury-induced mono-neuropathic pain. In fact, to have the full picture, future studies are needed to elucidate the antiallodynic effect of the test compounds at different doses and possible other mechanisms of action. Finally examining the effect of the combination following chronic treatment applied to the present and other animal models of NP would further justify the efficacy of the combination of tolperisone and pregabalin.

## 4. Materials and Methods

### 4.1. Animals

In the current study, 120–150 g male Wistar rats underwent partial sciatic nerve ligation (pSNL), and 170–200 g male Wistar rats were used for the rotarod assay and charcoal meal test to be matched with the operated animal’s body weights on the test days. Animals were purchased from Toxi-Coop Zrt. (Budapest, Hungary) and housed in standard cages of up to 4 or 5 animals/cage based on their weights, maintained at a controlled temperature (20 ± 2 °C), light/dark cycle (12/12 h), and allowed free access to food and water in the local animal house of Semmelweis University, Department of Pharmacology and Pharmacotherapy (Budapest, Hungary). All procedures and housing conditions were performed according to the European Communities Council Directives (2010/63/EU), the Hungarian Act for the Protection of Animals in Research (XXVIII.tv. 32.§), and the local animal care committee (PEI/001/276-4/2013 and PE/EA/619-8/2018).

### 4.2. Chemicals

Tolperisone and pregabalin were kindly provided as a gift by Meditop Pharmaceuticals Ltd. (Budapest, Hungary). Streptozotocin (STZ), carbamazepine, and duloxetine were purchased from Sigma–Aldrich (St. Louis, MO, USA). From Sigma–Aldrich, hydroxy ethyl cellulose, as well as glutamate oxidase, horseradish peroxidase, and Amplex Red for glutamate release measurement were purchased (St. Louis, MO, USA). All compounds were stored and handled according to manufacturing procedures.

### 4.3. Experimental Protocols of the Animal Study

Experimental schedule 1 displays a schematic overview of the experimental techniques used in this work. Baseline measurements were carried out by DPA (dynamic plantar esthesiometer 37450; Ugo Basil, Gemonio, Italy) to evaluate paw withdrawal thresholds before the operation, and animals then underwent pSNL surgery (see Section 4.4). On day 7 after the operation, baseline measurements were taken once more to test the development of mechanical allodynia, and neuropathic rats were then treated with compounds or vehicles. Mechanical allodynia was tested once more at 60, 120, and 180 min after acute oral administration to investigate the acute antiallodynic effect of the test drugs. In the chronic experiments, rats were given daily treatments for 14 days to assess the chronic antiallodynic impact of the investigated compounds, and then DPA was carried out on days 14 and 21, respectively, after surgery.

In another set of animals, baseline measurements were carried out by DPA, and pSNL surgery was performed. On day 14 after the operation, baseline measurements were taken once more to test the development of mechanical allodynia, and neuropathic rats were then treated with compounds or vehicles. Mechanical allodynia was tested once more at 60, 120, and 180 min after acute oral administration to investigate the acute antiallodynic effect of the test drugs and their combinations (Experimental schedule 2).

**Scheme 1:**
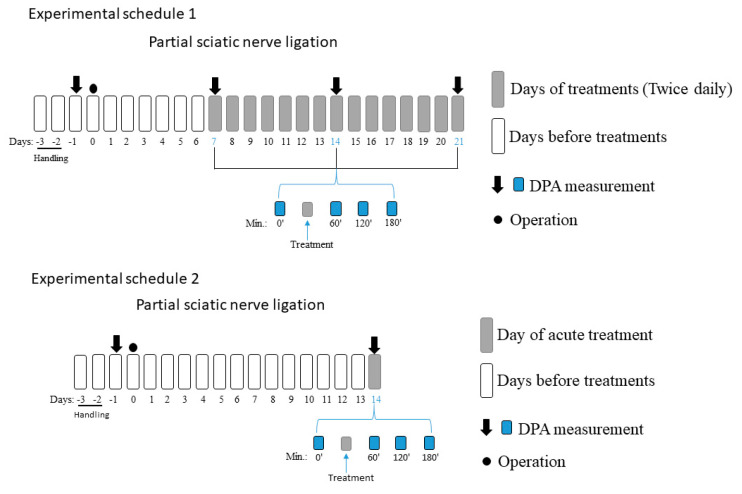
Experimental schedule 1.

Experimental schedule 1 (Scheme 1) shows the antiallodynic effects of pregabalin and tolperisone (both at 25, 50, and 100 mg/kg), carbamazepine (16.25, 32.5, and 65 mg/kg), and duloxetine (10 and 20 mg/kg) in rats that underwent partial sciatic nerve ligation evoked tactile allodynia measured by a DPA (dynamic plantar esthesiometer). In addition, the treatment day timeline and the precise intervals during the treatment days for DPA measurements are shown. Experimental schedule 2 shows the acute antiallodynic effects of pregabalin, tolperisone (both at 25, 50, and 100 mg/kg), their combination (25 mg and 25 mg), duloxetine (10 and 20 mg/kg), and the tolperisone and duloxetine combination (25 mg and 20 mg) in rats that underwent partial sciatic nerve ligation evoked tactile allodynia measured by DPA. The treatment day timeline and the precise intervals during the treatment days for DPA measurements are also shown.

### 4.4. Partial Sciatic Nerve Ligation (pSNL)

This was performed as previously described [33,77] for partial ligation of the sciatic nerve (pSNL). In summary, pentobarbital (i.p., 60 mg/kg, in a volume of 2.5 mL/kg) was used to produce anesthesia on the day of the operation, and rats were then placed on a pillow at 30 °C. The right dorsal back was shaved, an incision was created, and the sciatic nerve was carefully exposed in an aseptic setting. A size 7-0 polypropylene wire was then used to tightly ligate the exposed nerve at the level of the thigh, ensuring that the dorsal 1/3 to 1/2 of the nerve thickness was ligated. The wound was then closed with two stiches. In the sham group (controls), the sciatic nerve was exposed but not ligated.

### 4.5. Assessment of Mechanical Allodynia

Mechanical allodynia, the main symptom of neuropathic pain, was measured using the DPA, as previously mentioned [28,77]. Handling was performed in the days preceding the beginning of the experiments to acclimatize the animals to the experimental conditions by putting them in the plastic cages of the experimental setup once a day. The paw withdrawal thresholds (PWTs) of the animals were measured in grams (g). PWT values were assessed following a 5 min cage acclimatization period for each measurement. According to the manufacturer’s instructions, a metal filament with a diameter of 0.5 mm is raised alternately to the right and left hind paws (incrementation: 10 g/s, maximal force: 50 g). Three PWT measurements were performed on each paw, and the average of the three readings was calculated. For each animal, allodynia was defined as a 20% decrease in the average PWT value of the operated (right) paw compared to the unoperated (left) paw [28,38]. Measurements were performed in accordance with instructions in Section 4.3 or experimental schedules 1 and 2.

### 4.6. Treatment of Neuropathic Animals

The effects of pregabalin and tolperisone (both at 25, 50, and 100 mg/kg), duloxetine (10 and 20 mg/kg), and carbamazepine (16.25, 32.5, and 65 mg/kg) were investigated on day 7 after pSNL and assessments of allodynia began 60, 120, and 180 min after administration. The chronic treatments continued for 1–2 weeks, and again, assessment of the allodynia was determined on the day 14 and 21 after the operation. All drugs were administered twice per day in the chronic treatment experiments. In addition, the effects of pregabalin and tolperisone (both at 25, 50, and 100 mg/kg), duloxetine (10 and 20 mg/kg), the tolperisone and pregabalin combination (both at 25 mg/kg), and the tolperisone and duloxetine combination (25 mg/kg and 20 mg/kg), as well as carbamazepine (16.25, 32.5, and 65 mg/kg), were investigated after a single oral dose on day 14 after pSNL. The solution of all drugs was prepared in 0.9% saline, except for carbamazepine, which was suspended in 1% hydroxyethyl cellulose solution. All drugs were administered in a volume of 5 mL/kg via an orogastric gavage.

### 4.7. Motor Function Test

The rotarod test (Rat Rotarod, Model 7750; Ugo Basile, Gemonio, Italy) was used to evaluate the effect of test drugs on motor coordination in naïve rats. One day prior to the experiment, animals were trained to stay on the rotating rod of the apparatus for 180 s (cut-off time) where the instrument’s speed was adjusted to 16 rpm. On the following day, the acute effects of tolperisone (100 and 150 mg/kg), pregabalin (25, 50, and 100 mg/kg), and the tolperisone and pregabalin combination (both at 25 mg/kg), or vehicle, were tested after oral treatment at the time of the peak effect of the test drugs (60 and 120 min). The fall-off time, or the latency time, was recorded as an indication of motor coordination [10,78].

### 4.8. Determination of GastroIntestinal Peristalsis in Rats

The charcoal meal test was utilized to test the effect of tolperisone (25 and 50 mg/kg), pregabalin (25 and 50 mg/kg), or the tolperisone and pregabalin combination (both at 25 mg/kg) on gastrointestinal transit in rats after oral treatment [79]. In brief, naïve male Wistar rats were given free access to water and fasted for 18 h before the experiment. Drugs were administered, and 30 min later, an oral charcoal suspension (10% charcoal in 5% gum Arabic) in a volume of 2 mL/animal was given via oral gavage. After another 30 min, the rats were euthanized to take the whole small intestines. The charcoal travel distance was measured and compared to the whole small intestinal length.

### 4.9. Animal Model of Type 1 Diabetes-Induced Polyneuropathic Pain

For the STZ-induced type 1 diabetes model, male Wistar rats weighing 200–230 g were used. Animals were housed in a mesh-bottomed cage (type IV cage) that meets the EU’s requirement. To induce diabetes, we used a single intraperitoneal injection of 60 mg/kg of STZ, freshly dissolved in cold distilled water (1–3 °C) right before injection to prevent any degradation [80,81]. Three days later, diabetes was confirmed by measuring the blood glucose level (>14 mmol/L) in the blood obtained from the tail vein using the Dcont Etalon blood glucose meter (Roche Diagnostics GmbH, Mannheim, Germany). The highest blood glucose level that may be measured using a blood glucose test is 33.3 mmol/L [82]. Every third week, the PWTs were measured and expressed in g. Each hind paw’s PWT was measured three times alternatively. The average PWT values for each animal’s two paws were then determined. Age-matched (i.e., animals with age-matched to diabetic ones) and vehicle-treated groups were utilized as controls. An animal was considered neuropathic when the PWT value decreased by at least 20% compared to age-matched animals [38].

### 4.10. Capillary Electrophoresis Analysis of CSF Glutamate Content

In our lab, a modified technique of capillary electrophoresis laser-induced fluorescence detection was established [83] for assessing the glutamate level in CSF samples. At 14 days after the pSNL, neuropathic and control rats were sacrificed with isoflurane. CSF samples were obtained by puncturing the cisterna magna, centrifuged at 2000× *g* and 4 °C for 10 min, and deproteinized by combining with two volumes of cold acetonitrile and centrifuging at 20,000× *g* for 10 min at 4 °C. Supernatants were derivatized using NBD-F (1 mg/mL final concentration) in 20 mM borate buffer pH 8.5 for 20 min at 65 °C. As an internal standard, 1 µM L-cysteic acid was utilized. A P/ACE MDQ Plus capillary electrophoresis system with a laser-induced fluorescence detector adjusted to 488 and 520 nm excitation and emission wavelengths, respectively (SCIEX, Framingham, MA, USA), was used to evaluate derivatized materials. Separations were performed in polyacrylamide-coated fused silica capillaries (i.d.: 75 µm, effective/total length: 40/50 cm) at 15 °C with a constant voltage of −27 kV while using a 50 mM HEPES buffer pH 7.0 containing 6 mM 6-monodeoxy-6-mono (3-hydroxy) propylamino-β-cyclodextrin.

### 4.11. Glutamate Release from Synaptosomes

To examine the effects of tolperisone, pregabalin, or their combination on depolarization-evoked glutamate release, rat brain synaptosomes were prepared using a modified Modi et al. method [84]. In summary, animals were promptly decapitated, and their brains were separated and homogenized in a solution of 0.32 M sucrose and 4 mM HEPES (pH 7.4). After homogenate centrifugation (2 × 10 min, 1500× *g*, 4 °C), supernatants were collected and combined. After centrifuging the supernatant (2 × 10 min, 20,000× *g*, 4 °C), the pellet was resuspended in a buffer solution containing 4 mM HEPES, 0.32 M sucrose, 10% fetal bovine serum, and 10% dimethyl sulfoxide (DMSO), and stored at −80 °C until use. Glutamate release experiments were performed using a method described previously in our earlier publication [27].

On the experimental day, synaptosomal suspensions were defrosted, centrifuged (10 min, 20,000× *g*, 4 °C), and the pellet was resuspended in 10 mM HE-PES buffer containing 5.4 mM KCl, 130 mM NaCl, 0.9 mM MgCl_2_, 1.3 mM CaCl_2_, and 5.5 mM glucose (pH 7.4). The supernatant was collected from 10 mg synaptosomal suspensions centrifuged to an 8-well strip plate (15 min, 2500× *g*, 4 °C). Synaptosomes were equilibrated for 2 × 10 min at 37 °C before stimulation in HEPES buffer containing 40 µM DL-TBOA, a competitive, non-transportable blocker of excitatory amino acid transporters [23], to prevent glutamate reuptake. In the experiments, test drugs were added during the equilibration periods as pretreatment. After equilibration, a stimulation buffer containing 1 mM 4-aminopyridine was used to induce depolarization and subsequent glutamate release. Following stimulation, aliquots were taken at 8 min and stored at −20 °C until enzyme-linked fluorescent assay analysis.

### 4.12. Enzyme-Linked Fluorescent Assay of Glutamate Released from Synaptosomes

Glutamate release was measured using Glutamate Oxidase Assay Kit purchased from Sigma–Aldrich (St. Louis, MO, USA) using an enzyme-linked fluorescent assay. Briefly, the samples were mixed with a working solution containing glutamate oxidase (0.04 U/mL), horseradish peroxidase (0.125 U/mL), and Amplex Red (50 µM) (final concentrations), and fluorescent readings were performed after 30 min incubation at 37 °C. Excitation and emission wavelengths were 530 nm and 590 nm, respectively.

### 4.13. Statistical Analysis

GraphPad Prism 8.0 Software (San Diego, CA, USA), a statistical analysis program, was used to analyze the data. All data were presented as mean ± standard error of means (S.E.M.). All data were analyzed by one-way or two-way ANOVA followed by Dunnett’s post-hoc test for multiple comparisons. Significant differences were considered if *p* < 0.05. ROUT analysis was performed to identify outliers, with a Q value = 0.5%

## 5. Conclusions

The current consensus from the present work is that the onset of action of pregabalin and tolperisone to produce an antiallodynic effect is 2 weeks after oral administration. We have demonstrated, for the first time, that the oral combination of tolperisone and pregabalin acutely produces analgesia against allodynia evoked by pSNL without motor or gastrointestinal transit-related adverse effects. Mechanistically, targeting both voltage-gated sodium and calcium channels could modulate the glutamatergic neurotransmission as reflected by the normalized neuropathy-induced elevation of the CSF glutamate content. The preclinical pharmacological characterization of existing and novel medications, or their combination, can provide clinical researchers with actionable and objective insights into developing or repurposing attitudes.

## Data Availability

Data is contained within the article.
